# Plasmonic spin induced Imbert–Fedorov shift

**DOI:** 10.1515/nanoph-2022-0787

**Published:** 2023-02-15

**Authors:** Hao You, Abdullah Alturki, Xiaodong Zeng, Muhammad Suhail Zubairy

**Affiliations:** Department of Physics, Shanghai University, Shanghai 200444, China; Institute for Quantum Science and Engineering (IQSE) and Department of Physics and Astronomy, Texas A&M University, College Station, USA

**Keywords:** Imbert–Fedorov shift, longitudinal plasmonic spin, spin–orbit coupling, surface plasmon polaritons on chiral material interfaces

## Abstract

The spin angular momentums of surface plasmon polaritons (SPPs) on chiral material interfaces and the Imbert–Fedorov shifts of linearly polarized light beams are investigated. Compared to a traditional TM-polarized SPP having a transverse spin, the SPP on a chiral material interface also has a longitudinal spin component, resulting from the nature that this new kind of SPP is a hybrid of TE and TM-polarized evanescent waves. When a light beam is incident on a sandwich structure composed of chiral material, prisms, and metal layers, in which the SPP is supported, the reflection and transmission processes can be analogous to the transport of a photon in a waveguide QED system. The SPP with longitudinal spin can be excited by the incident wave and the reflected and transmitted beams carry the spin features of the SPP. Moreover, the beams exhibit large Imbert–Fedorov shifts stemming from the spin–orbit coupling even for a linearly polarized incident beam. The shifts are determined by the longitudinal spin angular momentum and excitation coefficient of the SPP. This present work extends the study of photonic spin–orbit coupling and provides an important platform to investigate the plasmonic spin.

## 1 Introduction

Analogous to the spin Hall effect of electrons in a nonmagnetic environment, there exists a transverse shift (perpendicular to the plane of incidence) of the interfacial reflection of a light beam [1–9]. The shift, called Imbert–Fedorov shift, has a direction that depends on the photonic spin angular momentum (AM). To conserve the total angular momentum normal to the interface, the spin AM is partially converted to orbital AM. This results in the center shift of the reflected beam. This process due to the spin–orbit coupling has attracted extensive interest due to its promising applications in optical devices and fundamental physics [1]. Compared to the spin Hall effect in an electron system in which an electric field is applied, optical interface is required to realize the optical spin Hall effect. Normally, the spin–orbit coupling of light is weak and the Imbert–Fedorov shift of the beam is tiny in the sub-wavelength range. This makes it difficult to be observed [8, 9]. To enhance the effect, photonic structures with complex spatial dispersion can be constructed, such as photonic crystal, metamaterial, and metasurface [10–17]. In which, metal-based optical structures have been widely studied for the enhancement and regulation of the spin Hall effect because of their surface plasmon resonance effect [18–31]. Theories and experiments have shown that the surface plasmon polariton (SPP) can enhance the beam spin-splitting efficiently. The flexible tunability of plasmonic structures also expand the applications of spin–orbit coupling [1], such as in precision measurement [32] and spin optoelectronic devices [33]. In fundamental physics, the investigations of spin AM and spin–orbit coupling based on SPP also attracted tremendous interest in the past 10 years [34–39]. The SPP, even for the simplest SPP mode on a metal interface, possesses lots of unpredicted physics, such as nonzero spin energy flow and spin AM. Contrary to the circularly polarized field in free space with spin along the propagation direction, called longitudinal spin, the spin of the TM-polarized SPP is perpendicular to the propagation direction, called transverse spin [35]. By optical scattering of the evanescent wave with Mie particle, the evanescent wave spin AM can be converted to the Mie particle [36]. In turn, the unidirectional SPP can be excited by the interaction between Mie particle and circularly polarized optical wave [37].

Chiral materials exist widely in nature [40]. For instance, many biomolecules exhibit strong chirality. The chirality in atomic gas systems can even induce negative refraction [41]. In addition, strong chirality has been achieved artificially in many systems such as in nanoplasmonic structures [42, 43] and nanoparticle–chiral molecule hybrid structures [44, 45]. In bulk chiral material, optical waves propagate with spin-dependent wave numbers, i.e., optical rotatory dispersion effect. This effect has attracted great interest due to its potential applications, such as in nanophotonics and optical sensing [40].

On chiral material–metal or even chiral material–dielectric interfaces, there also exist SPPs [46, 47]. The SPP on the chiral material interface is a hybrid mode of TE and TM-polarized waves, or left- and right-circularly polarized waves but with asymmetric amplitudes. This means this kind of mode possesses electric fields in all three directions [46, 47].

In the present paper, we show that the two components of the electric field, i.e., the components perpendicular to plane of incidence and perpendicular to the interface, exhibit a *π*/2 phase difference, which demonstrates that the SPP on the chiral material interface not only has a transverse spin component similar to normal SPP but also a longitudinal spin component. Moreover, symmetric and antisymmetric modes can be found on a chiral material slab with suitable parameters. On the two interfaces, both kinds of modes have same longitudinal but opposite transverse spin density. This results in a total zero transverse but nonzero longitudinal spin AM. To excite the SPP, a sandwich structure is introduced, which composes of chiral material slab, prisms, and metal layers. When a TE or TM-polarized light beam with zero spin is incident on the structure, it interacts with the TE or TM-polarized components of the SPP, and the SPP can be excited under phase-matching conditions. The reflection and transmission of the incident wave through the sandwich structure can be analogous to a photon transport in a waveguide quantum electromagnetic dynamical system (waveguide QED) [48, 49], in which the SPP acts as the chiral scatter. As a consequence of the nonzero longitudinal spin of the SPP, the reflected and the transmitted beams can possess nonzero spins as well as large transverse shifts. The shift resulting from the plasmonic spin–mediated spin–orbit coupling is directly related to the longitudinal spin AM density and excitation coefficient of the SPP. The present investigation can provide an important platform to investigate the plasmonic spin as well as the spin–orbit coupling involving SPP, such as quantifying the plasmonic spin and measuring the chiral material parameters [50] by measuring the transverse shifts.

## 2 Spin AM density of SPP on a chiral material interface

**Figure 1:**
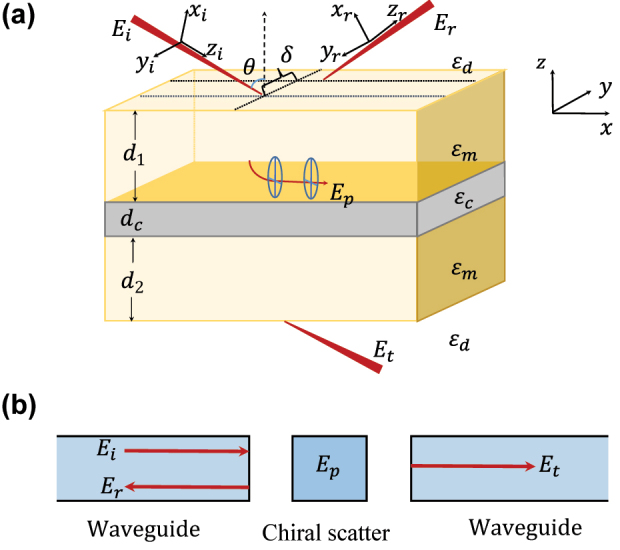
Light propagates in nanophotonic structures. (a) Multilayer structure supporting the hybrid SPP. From up to down, the layers are dielectric/metal/chiral material/metal/dielectric. The permittivities of the dielectric and metal are *ɛ*_*d*_ and *ɛ*_*m*_. The thicknesses of the metal layers are *d*_1_ = *d*_2_ = *d*_*m*_. The chiral material is described in Eqs. (1) and (2) and has a thickness *d*_*c*_. (b) Waveguide-chiral scatter-waveguide system. The propagation of optical wave can be analogous to the transport of a photon in a waveguide QED system [49], where the chiral scatter can be resonator or atom having different coupling strength with left- and right-circularly polarized fields [53].

Our model is schematically shown in Figure 1. The system consists in a multilayered heterostructure and the layers are assumed to be infinite in the *x* − *y* plane. The middle layer is a chiral material slab. To excite the SPP in the present configuration, the upper and bottom layers with large permittivity *ɛ*_*d*_, which act as prisms, are introduced. The chiral material is typically characterized by constitutive relations as [51]:

(1) $$D=\varepsilon_{c}\varepsilon_{0}E+i\frac{\kappa }{c}H,$$

(2) $$B=\mu_{c}\mu_{0}H-i\frac{\kappa }{c}E.$$

Here, *κ* is the chirality parameter, *c* is the speed of light in vacuum, *ɛ*_0_ and *μ*_0_ are the vacuum permittivity and permeability, *ɛ*_*c*_ and *μ*_*c*_ = 1 are the relative permittivity and permeability, respectively. In the following calculations, we set *ɛ*_*c*_ = 4 and *κ* = 0 ∼ 0.5. The strong chirality can be realized by dense DNAs or proteins. These molecules do not absorb from visible to near-infrared wavelength intervals [44, 45]. Inside the chiral medium, right- and left-circularly polarized waves propagate with wave number denoted by *k*_+_ and *k*_−_, where *k*_±_ = *k*_0_*n*_±_ = *k*_0_(*n*_*c*_ ± *κ*) with *k*_0_ = *ω*/*c* being the vacuum wave number and *ω* the harmonic frequency. By taking into account the continuities of the electric field **E** and magnetic field **H** along both *x* and *y* directions, it is straightforward to derive the dispersion relation of the SPP as shown in Eq. (S26) of the Supplementary material. In the sandwich configuration, the SPP mode of interest is a leaky mode [52]. As a consequence, $n_{+}k_{0}<\text{Re}\left(k_{p}\right)<\sqrt{\varepsilon_{d}}k_{0}$ and *k*_*p*_ has an imaginary part resulting from the leakage as well as the intrinsic loss of the layers.

**Figure 2:**
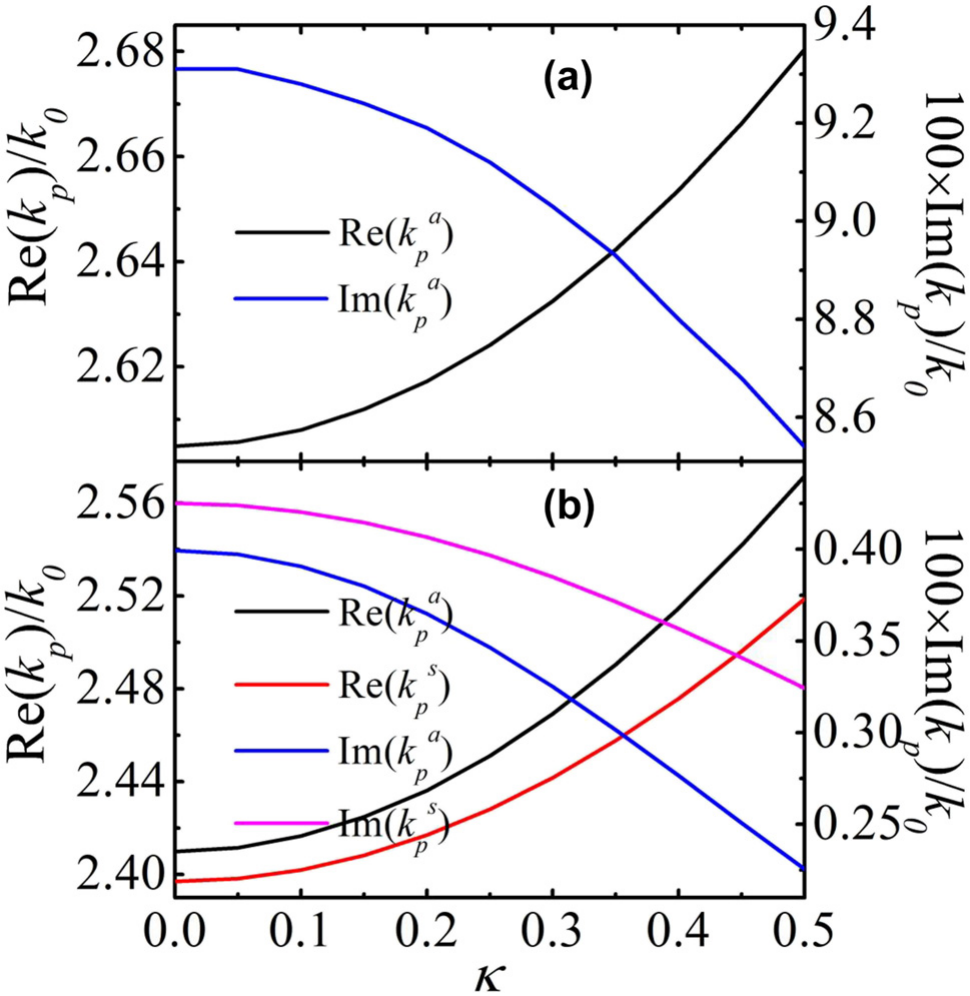
The dispersion relation of the wave number of the SPP as a function of the chirality. Here, *λ*_0_ = 1060 nm, *ɛ*_*c*_ = 4, *ɛ*_*m*_ = −13 + 0.1*i*, *ɛ*_*d*_ = 11.9. (a) *d*_*c*_ = 200 nm and *d*_*m*_ = 50 nm. (b) *d*_*c*_ = 700 nm and *d*_*m*_ = 250 nm.

In Figure 2, the plasmonic wave numbers as a function of *κ* are presented. It shows that, as the chirality increases, the real parts of the wave number increase slightly and the imaginary parts decrease. When the chiral material layer is thin (*d*_*c*_ = 200 nm), only one asymmetric mode is supported, where the charge carrier densities on the two interfaces of the chiral material are out of phase. However, when the layer is thick (*d*_*c*_ = 700 nm), a symmetric mode with a smaller wave number appears. Here, the charge carrier densities on the two interfaces of the chiral material are in phase. Since the metal thickness determines the leak rate of the SPP, the imaginary part of the plasmonic wave number is much larger for a smaller *d*_*m*_.

In the dispersion relation of Eq. (S26), the reflectivities of the interface from the metal to the prism for both TE and TM-polarized waves are approximated to be 1. When the metal layer thickness is large (for example *d*_*m*_ > 100 nm), $e^{-2\text{Im}\left(\beta_{m}\right)d_{m}}\ll 1$ , where $\beta_{m}=\sqrt{\varepsilon_{m}k_{0}^{2}-k_{p}^{2}}$ . It is convenient to achieve that the dispersion relation of the sandwich configuration as shown in Figure 1 can be almost the same as that of a chiral slab embedded in infinite metal. For example, the plasmonic wave number Re(*k*_*p*_) = 2.5432  *k*_0_ when *d*_*c*_ = 1000 nm and *d*_*m*_ = 250 nm while Re(*k*_*p*_) = 2.5433  *k*_0_ when the metals are semi-infinite.

**Figure 3:**
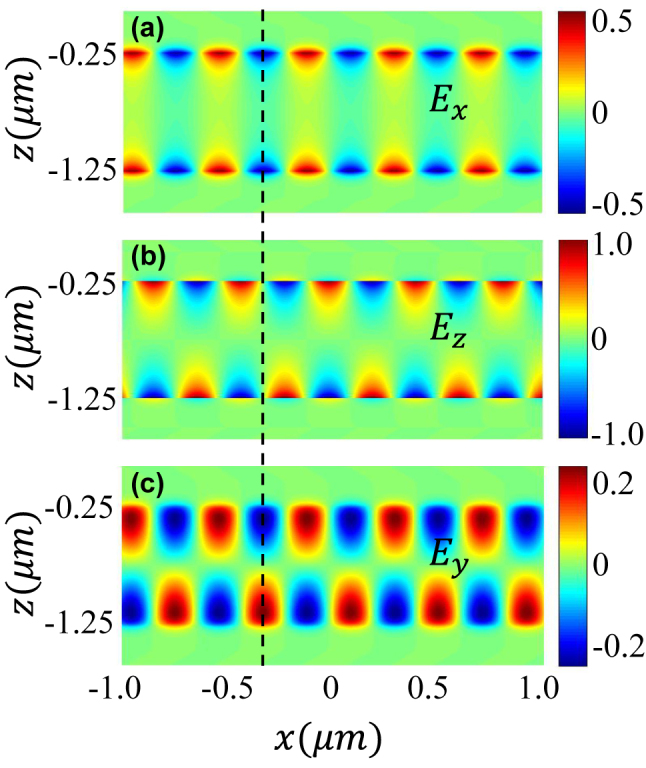
The electric field distribution of the symmetric mode for all three directions. The field amplitudes are normalized by the maximum value of *z* direction component. Here, *d*_*c*_ = 1000 nm, *d*_*m*_ = 250 nm, and *κ* = 0.5.

The field distribution of the symmetric SPP mode is presented in Figure 3 (also see the field distribution of the asymmetric mode as Figure S1 in the Supplementary material). Compared to the usual TM-polarized SPP having electric fields along the *x* direction and *z* direction, the SPP in the present model has an additional electric field component along *y* direction. The figure shows that, for both the symmetric and asymmetric modes, the *y* direction electric field has the same phase with the *x* direction while *π*/2 phase difference with the *z* direction electric field. Moreover, compared to the *z* and *x* direction electric fields concerted on the interfaces, the *y* direction electric field distributes on the whole chiral material more uniformly. The electric field of the symmetric mode for infinite *d*_*m*_ is shown in Figure S2 in the Supplementary material. The field distribution near the chiral material interfaces is almost the same as Figure 3.

In the metal, the electric field of the SPP can be expressed as the coherent summation of “left-circularly polarized” and “right-circularly polarized” fields as [47]

(3) $$E\approx a{\hat{E}}_{L}+b{\hat{E}}_{R},$$

where in the upper metal layer

(4) $${\hat{E}}_{R/L}=\frac{1}{\sqrt{2}}\left(\frac{\beta_{m}}{k_{m}}\hat{x}-\frac{k_{p}}{k_{m}}\hat{z}\pm \hat{y}\right)e^{ik_{p}x+i\beta_{m}\left(z+d_{m}\right)}{\text{e}}^{-\text{i}\omega t}.$$

Here, *a* and *b* have real but unequal values. Since both the left- and right-circularly polarized components have the same wave numbers *k*_*p*_, the *x* and *z* components of the SPP electric field are identical to a normal TM-polarized evanescent wave. All three components of the electric field decay exponentially as $e^{-\text{Im}\left(\beta_{m}\right)d}$ away from the chiral slab, where *d* is the distance to the chiral slab.

In a dispersive material with no chirality, the spin AM density of an optical wave is given by [36]

(5) $$s=\text{Im}\left(\frac{d\left(\omega \varepsilon \varepsilon_{0}\right)}{d\omega }E^{*}\times E+\frac{d\left(\omega \mu \mu_{0}\right)}{d\omega }H^{*}\times H\right)/4\omega ,$$

where *ɛ* and *μ* are the frequency dependent permittivity and permeability, respectively. In the dielectric, *d*(*ωɛ*)/*dω* ≈ *ɛ*_*d*_, while in metal, *d*(*ωɛ*)/*dω* ≈ 2 − *ɛ*_*m*_, in which the metal has a Drude type permittivity $\varepsilon_{m}=1-\omega_{p}^{2}/\omega \left(\omega +i\gamma \right)$ with *γ* as the scattering rate. We assume that in both kinds of materials, *d*(*ωμ*)/*dω* = 1. In a chiral material, such as the chiral slab in Figure 1(a), **s** can be modified to

(6) $$\begin{array}{ll} s & =\text{Im}\left(\frac{d\left(\omega \varepsilon_{c}\varepsilon_{0}\right)}{d\omega }E^{*}\times E+i\frac{d\left(\omega \kappa \right)}{cd\omega }E^{*}\times H\right.\\ & \;\left.+\frac{d\left(\omega \mu_{c}\mu_{0}\right)}{d\omega }H^{*}\times H-i\frac{d\left(\omega \kappa \right)}{cd\omega }H^{*}\times E\right)/4\omega . \end{array}$$

In the following calculations, we assume the *ɛ*_*c*_, *μ*_*c*_, and *κ* are weakly dispersive at the chosen frequency, i.e., *d*(*ωɛ*_*c*_)/*dω* ≈ *ɛ*_*c*_, *d*(*ωμ*_*c*_)/*dω* ≈ *μ*_*c*_, and *d*(*ωκ*)/*dω* ≈ *κ*. Using the electromagnetic fields defined in Eq. (3), the spin AM density in the metal can be expressed as

(7) $$s_{x}\approx \text{Im}\left[\frac{\varepsilon_{0}\left(a^{2}-b^{2}\right)k_{p}}{2\omega k_{m}}\right]e^{-2\text{Im}\left(\beta_{m}\right)\left(z+d_{m}\right)},$$

(8) $$\begin{array}{ll} s_{y} & \approx \text{Im}\left\{\frac{\left[\left(2-\varepsilon_{m}\right){\left(a+b\right)}^{2}+{\left(a-b\right)}^{2}\varepsilon_{m}\right]\varepsilon_{0}\beta_{m}k_{p}}{4\omega k_{m}^{2}}\right\}\\ & \;\times e^{-2\text{Im}\left(\beta_{m}\right)\left(z+d_{m}\right)}, \end{array}$$

(9) $$s_{z}\approx 0.$$

**Figure 4:**
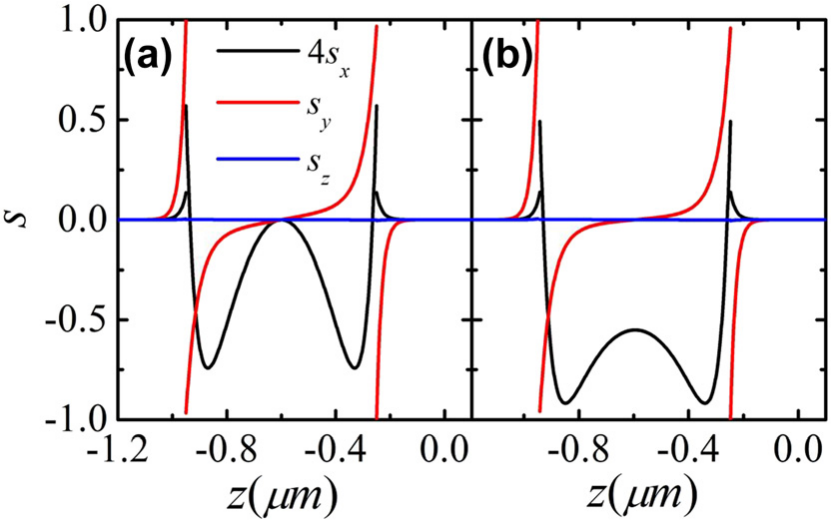
The normalized spin AM densities of the symmetric (a) and antisymmetric modes (b) along three directions. The parameters are the same as Figure 2(b).

The spin AM of the SPP can be calculated by $S=\int_{-\infty }^{\infty }sdr$ [36]. We plot the spin AM density of the SPP versus *z* for all three direction components under typical parameters in Figure 4. The spin density is not continuous on the chiral slab interfaces and agrees with the approximated results of Eqs. (7)–(9) very well. Since the *x* and *y* electric fields are phase synchronization, the *z* component spin AM density is approximately equal to 0 for both symmetric and asymmetric modes. Meanwhile, the spin AM density has the same values on the two interfaces for the *x* component, i.e., the longitudinal spin, but the opposite values for the *y* component, i.e., the transverse spin. This means that the total transverse spin AM of the SPP vanishes. The nonzero longitudinal spin AM demonstrates that when a linearly polarized incident field couples to the mode, the reradiated field can carry the spin feature of the SPP. In the metal, the spin AM density decays away from the chiral slab with an exponential rate two times larger than that of the electric field. Compared to a TM-polarized SPP with plasmonic wave number *k*_*p*_, the *y* component spin AM density has the contribution from magnetic field.

## 3 Plasmonic spin enhanced Imbert–Fedorov shifts of linearly polarized beams

In the following, we show that longitudinal spin of the SPP dominates the interaction between an incident field and the structure as shown in Figure 1(a). In paraxial optics, the incident Gaussian light beam can be written as [51]:

(10) $$E_{i}\left(x_{i},y_{i},z_{i}\right)=\left(f_{p}{\hat{e}}_{ix}+f_{s}{\hat{e}}_{iy}\right)e^{-\frac{k_{d}}{2}\frac{x_{i}^{2}+y_{i}^{2}}{Z_{R}+iz_{r}}},$$

where *Z*_*R*_ = *k*_d_*W*^2^/2 is the Rayleigh length and *W* is the beam waist. The complex-valued *f*_*p*_ and *f*_*s*_ determine the polarization of the beam. ${\hat{e}}_{iy}$ and ${\hat{e}}_{iy}$ denote the unit vectors of the electric fields of TM and TE-polarized waves, respectively. The electric field of the beams can be expressed in terms of their angular spectrum as follows

(11) $$\begin{array}{ll} & E_{j}\left(x_{j},y_{j},z_{j}\right)\\ & \;=\int E_{j}\left(k_{jx},k_{jy}\right){\text{e}}^{\text{i}\left(k_{jx}x_{j}+k_{jy}y_{j}+k_{jz}z_{j}\right)}dk_{jx}dk_{jy}. \end{array}$$

Here, *j* = *i*, *r*, *t* refer to the incident, reflected, and transmitted fields. *k*_*jx*_ and *k*_*jy*_ are the wave vector components in the corresponding *j* coordinates. For a Gaussian beam with a large waist, $k_{iz}\approx k_{d}-\left(k_{ix}^{2}+k_{iy}^{2}\right)/2k_{j}$ by keeping the square root of *k*_*iz*_ to the first order.

The angular spectrums are related by the electromagnetic boundary conditions on the interfaces as shown in Section 1 of the Supplementary material. The reflected field can be obtained as

(12) $$\begin{array}{ll} \left(\begin{array}{c} E_{rp}\\ E_{rs} \end{array}\right) & =e^{-\frac{Z_{R}\left(k_{rx}^{2}+k_{ry}^{2}\right)}{2k_{d}}}\\ & \;\times \left(\begin{array}{c} r_{\mathrm{pp}}+\frac{k_{ry}R_{2}}{k_{d}}\cot\theta \;r_{ps}+\frac{k_{ry}R_{1}}{k_{d}}\cot\theta \\ r_{\mathrm{sp}}-\frac{k_{ry}R_{1}}{k_{d}}\cot\theta \;r_{ss}+\frac{k_{ry}R_{2}}{k_{d}}\cot\theta \end{array}\right)\left(\begin{array}{c} f_{p}\\ f_{s} \end{array}\right) \end{array}$$

**Figure 5:**
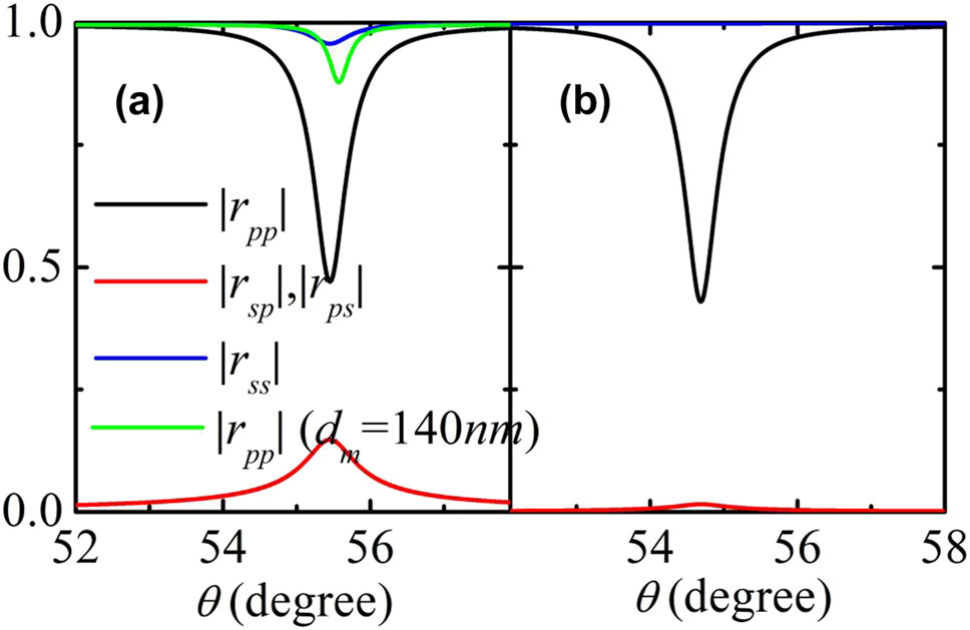
The reflections of the structure with chirality (a) *κ* = 0.5 and (b) *κ* = 0.05. Here, *d*_*m*_ = 100 nm and *d*_*c*_ = 150 nm.

Here, *R*_1_ = *r*_*ss*_ + *r*_*pp*_, *R*_2_ = 2*r*_*sp*_. *r*_*ij*_ (*i*, *j* = *s*, *p*) are the Fresnel reflection coefficients, in which *j* denotes the polarization of the incident wave while *i* refers to the polarization of the reflected wave. *θ* is the incident angle. According to Eq. (12), all the influences of the SPP structure on the incident wave are characterized by *r*_*ij*_. In Figure 5, the |*r*_*ij*_| at different *κ* are presented. As shown in Figure 3, the TE-polarized component has a relatively small proportion of the total energy of the SPP even for a large *κ*, which induces an almost unchanged exciting probability of SPP for a TM-polarized incident wave since it only interacts with the TM component of the SPP. As a consequence, *r*_*pp*_ varies slightly by changing *κ* and almost equal to the case that *κ* = 0. On the contrary, *r*_*sp*_ is directly proportional to *κ*. Additionally, for TE-polarized incident waves, the exciting probability is much lower than those of TM-polarized incident waves and consequently, the reflection of TE-polarized incident wave is almost equal to 1.

In physics, the reflection and transmission of the incident wave can be analogous to the transport of a photon in a waveguide QED system as shown in Figure 1(b) [48, 49]. The SPP plays the role of a chiral scatter [53] with tunable chirality, i.e., plasmonic spin, which can be manipulated by controlling the *d*_*c*_, *ɛ*_*c*_, *κ*, *d*_*m*_, and other parameters. As shown in Figure 1(b), when the photon hits the right end of the left waveguide, it can be reflected with reflection denoted by *r*^*D*^. The photon also interacts with the nearby resonator via near field coupling. For far detuning case, i.e., the photon has a large frequency difference with the resonator, the coupling can be neglected and the photon can be reflected with a unit probability. For the resonant case, the resonator can be efficiently excited and the radiation field to the left waveguide, denoted by *r*^*R*^, has *π* phase difference with *r*^*D*^. The destructive interference between *r*^*D*^ and *r*^*R*^ weakens the total reflection and the transmission is enhanced since the excited resonator can radiate to the right waveguide. As a consequence, the near field coupling strength and detuning determine the reflection and transmission probabilities.

In the present model as shown in Figure 1(a), when a light beam propagating in the upper dielectric is incident on the structure, it can be reflected by the interface between the dielectric and the metal with a coefficient *r*^*D*^. For a TM-polarized beam, $r_{p}^{D}=\frac{\varepsilon_{m}\beta_{d}-\varepsilon_{d}\beta_{m}}{\varepsilon_{m}\beta_{d}+\varepsilon_{d}\beta_{m}}$ . While for a TE-polarized beam, $r_{s}^{D}=\frac{\beta_{d}-\beta_{m}}{\beta_{d}+\beta_{m}}$ . If there is no SPP supported in the structure, the above reflection coefficients are almost equal to the total coefficients and can be almost unity. However, when a SPP exists, it can be excited via near field coupling. Compared to the waveguide QED system, where the strong coupling requires the frequency resonance, strong coupling in the present multilayered configuration needs phase (wave vector) matching, i.e., the incident angle *θ* satisfies $\sqrt{\varepsilon_{d}}\sin\theta =k_{p}$ . After the SPP is excited, it radiates its TM (TE) components to TM (TE)-polarized reflected fields. The coupling strength between the incident (reflection) fields and the SPP determines the excitation (radiation) ability. Since the SPP has different electric field intensities along *x* and *y*, the corresponding coupling between TM or TE-polarized propagating waves and the SPP has different strengths, which can be denoted by Ω_*p*_ and Ω_*s*_, respectively. Both *r*_*ps*_ and *r*_*sp*_ are proportional to Ω_*p*_Ω_*s*_ and as a consequence have the same amplitudes, which agree well with the numerical results as shown in Figure 5. Considering the electric field directions of TE and TM-polarized fields, it is convenient to obtain *r*_*sp*_ = −*r*_*ps*_. This result can also be easily understood by the time reversal symmetry effect.

As Ω_*p*_ and Ω_*s*_ are negatively related to *d*_*m*_ exponentially, the reflection coefficients are strongly dependent on the metal thickness. As shown in Figure 5(a), the reflection has a lager amplitude for a thicker metal layer. For a certain *d*_*m*_, *r*_*pp*_ has an approximated formulation as:

(13) $$r_{\mathrm{pp}}\approx R_{\mathrm{pp}}=r_{p}^{D}-\left(1+r_{p}^{D}\right)E_{p}^{xm}{\text{e}}^{\text{i}\beta_{m}d_{m}}k_{d}/\beta_{d},$$

In the above expression, the amplitude of the incident field is set to 1. Here, $E_{j}^{am}$ are the excited plasmonic electric fields at *z* = −*d*_*m*_ in the metal side for *a* = *x*, *y* directions, in which *j* = *s*, *p* denote TE and TM-polarized incident fields, respectively. $E_{p}^{xm}\propto r_{pp}-r_{p}^{D}$ and we denote $r_{pp}-r_{p}^{D}$ as the excitation coefficient of the SPP, which is weakly dependent on the chirality *κ* as shown in Figure 5. The other reflection coefficients can be written as

(14) $$R_{\mathrm{sp}}=\left(1-r_{s}^{D}\right)E_{p}^{ym}{\text{e}}^{\text{i}\beta_{m}d_{m}},$$

(15) $$R_{ps}=-\left(1+r_{p}^{D}\right)E_{s}^{xm}{\text{e}}^{\text{i}\beta_{m}d_{m}}k_{d}/\beta_{d},$$

(16) $$R_{ss}=r_{s}^{D}+\left(1-r_{s}^{D}\right)E_{s}^{ym}{\text{e}}^{\text{i}\beta_{m}d_{m}}.$$

By using *r*_*sp*_ = −*r*_*ps*_ and the spin AM density defined in Eq. (7), it is straightforward to obtain all the other reflection coefficients as

(17) $$R_{\mathrm{sp}}=-R_{ps}=s_{x}\left(r_{pp}-r_{p}^{D}\right)\eta ,$$

(18) $$R_{ss}=r_{s}^{D}-s_{x}^{2}\left(r_{pp}-r_{p}^{D}\right)\eta^{2}.$$

**Figure 6:**
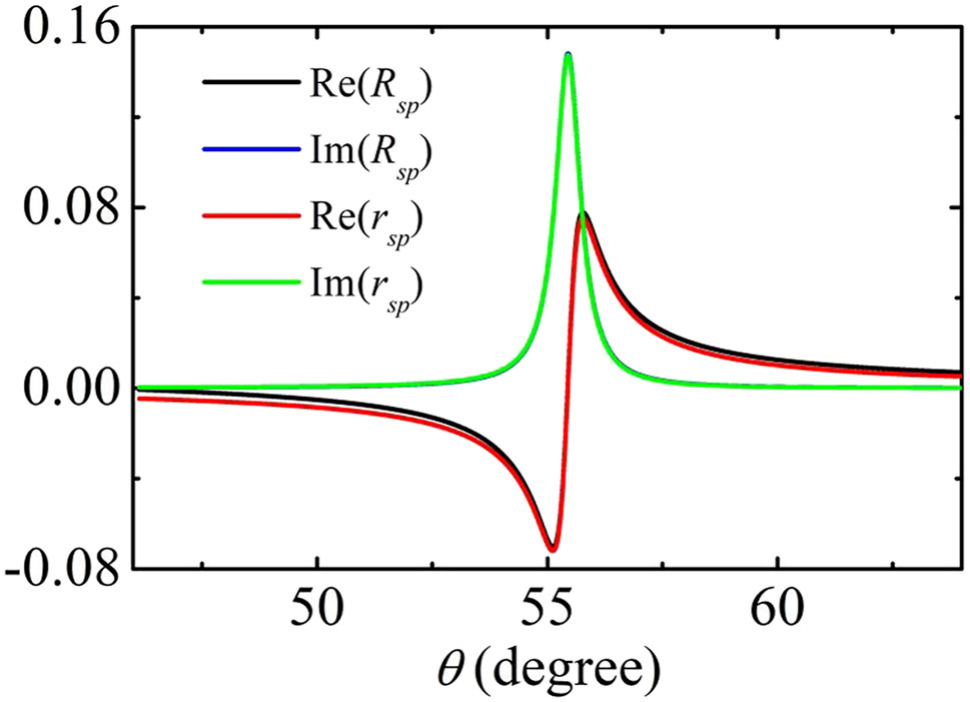
The comparison of reflection *r*_*sp*_ calculated by the method in Section 1 of the Supplementary material and *R*_*sp*_ calculated by Eq. (17). Here, *κ* = 0.5, *d*_*m*_ = 100 nm, and *d*_*c*_ = 150 nm.

Here, $\eta =i\omega \left(1-r_{s}^{D}\right)\beta_{d}k_{p}/\varepsilon_{0}\left(1+r_{p}^{D}\right)k_{d}\beta_{m}$ . *s*_*x*_ is calculated from Eq. (7) with $\left|E_{z}|=|-\left(a+b\right)k_{p}/\sqrt{2}k_{m}\right|$ set to be 1. Eq.(17) is the key result of this article. It is clearly shown that the reflection coefficients *R*_*sp*_ are proportional to the longitudinal spin AM densities *s*_*x*_ as well as the excitation coefficient of the SPP. In Figure 6, we show that the approximated results *R*_*ij*_ (*i*, *j* = *s*, *p*) and the exact calculations *r*_*ij*_ by the method in Section 1 of the Supplementary material are in good agreement. This proves the validity of the model as shown in Figure 1(b). The nonzero *s*_*x*_ means the reflected and transmitted fields are not linearly polarized anymore.

**Figure 7:**
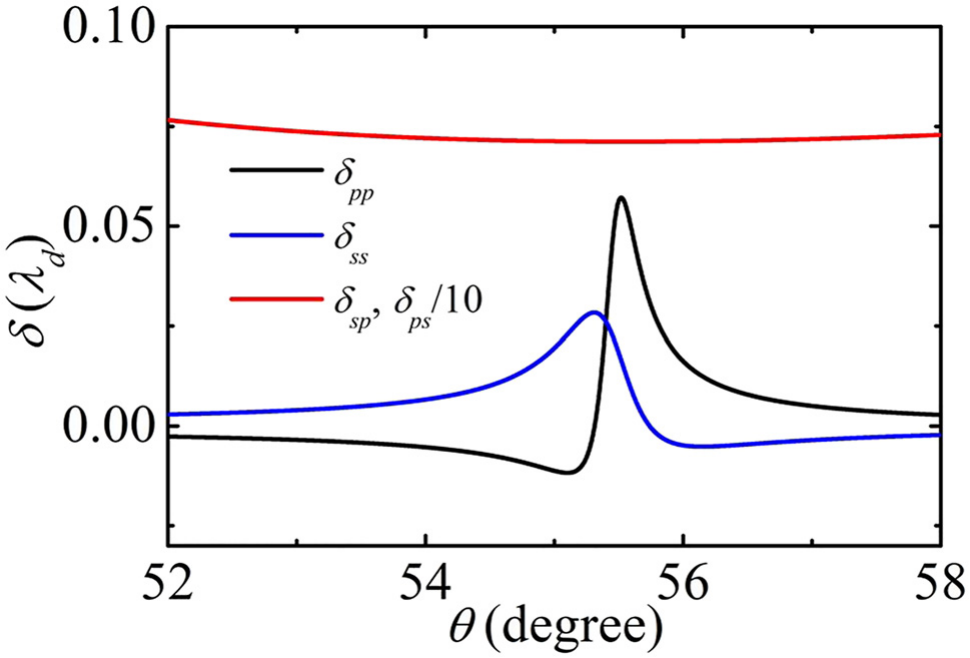
The transverse shifts *δ* of the linearly polarized reflected components by linearly polarized incident beams. The parameters are the same as Figure 6.

In the following, we consider the transverse shift of a Gaussian beam incident on the SPP structure. According to Eq. (12), for a TM-polarized incident beam, the reflection can be separated into TM and TE-polarized components as

(19) $$E_{r}\left(k_{rx},k_{ry}\right)=r_{pp}e^{ik_{ry}\Delta_{pp}}{\hat{e}}_{rx}+r_{sp}e^{ik_{ry}\Delta_{sp}}{\hat{e}}_{ry},$$

while for a TE-polarized incident beam,

(20) $$E_{r}\left(k_{rx},k_{ry}\right)=r_{ss}e^{ik_{ry}\Delta_{ss}}{\hat{e}}_{ry}+r_{ps}e^{ik_{ry}\Delta_{ps}}{\hat{e}}_{rx},$$

where Δ_*pp*_ = −*iR*_2_ cot *θ*/(*r*_*pp*_*k*_*d*_), Δ_*sp*_ = Δ_*ps*_ = *iR*_1_cot *θ*/(*r*_*sp*_*k*_*d*_), and Δ_*ss*_ = −*iR*_2_cot *θ*/(*r*_*ss*_*k*_*d*_). *δ*_*ij*_ = −Re(Δ_*ij*_) (*i*, *j* = *p*, *s*) are the transverse shifts for the *i* polarized reflected components of *j* polarized incident beams, which are related to *r*_*sp*_. The presence of *s*_*x*_ means the reflection field has a nonzero spin AM. The zero spin AM of the incident beam is split into the nonzero optical spin AM and orbit spin AM. Consequently, the transverse shift *δ*_*ij*_ can be explained by plasmonic spin–assisted spin–orbit coupling. As shown in Eq. (17), *r*_*sp*_ is proportional to *s*_*x*_. While as shown in Eq. (18), *r*_*pp*_ and *r*_*ss*_ are weakly related to *s*_*x*_. As a consequence, *δ*_*pp*_ and *δ*_*ss*_ are proportional to *s*_*x*_ while *δ*_*sp*_ is inverse proportional to *s*_*x*_. In Figure 7, we plot the shifts of linearly polarized reflected beams. At the incident angle *θ* satisfying *k*_*d*_ sin *θ* = *k*_*p*_, large transverse spatial shifts present. Note that when *κ* is small, |*r*_*sp*_| is tiny and *δ*_*sp*_ is large. This means a balance should be struck between the experimentally observable shift and the reflection probability.

The total transverse shift of the centroid of the reflected beam can be expressed as (see Section 3 in the Supplementary material):

(21) $$\left\langle y_{r}\right\rangle =\frac{\Delta_{ry}}{k_{d}\tau }+\frac{z_{r}}{Z_{R}}\frac{\delta_{ry}}{k_{d}\tau }.$$

Here

(22) $$\begin{array}{ll} \Delta_{ry} & =-\cot\theta \times Im\left[r_{pp}r_{ps}^{*}+r_{sp}r_{ss}^{*}+2\left(r_{pp}^{*}r_{sp}|f_{p}{|}^{2}\right.\right.\\ & \left.\;+r_{ps}^{*}r_{ss}|f_{s}{|}^{2}\right)-f_{p}f_{s}^{*}\left(|r_{pp}{|}^{2}+2r_{pp}r_{ss}^{*}+|r_{sp}{|}^{2}\right.\\ & \left.\left.\;-2r_{ps}^{*}r_{sp}+|r_{ps}{|}^{2}+|r_{ss}{|}^{2}\right)\right]. \end{array}$$

**Figure 8:**
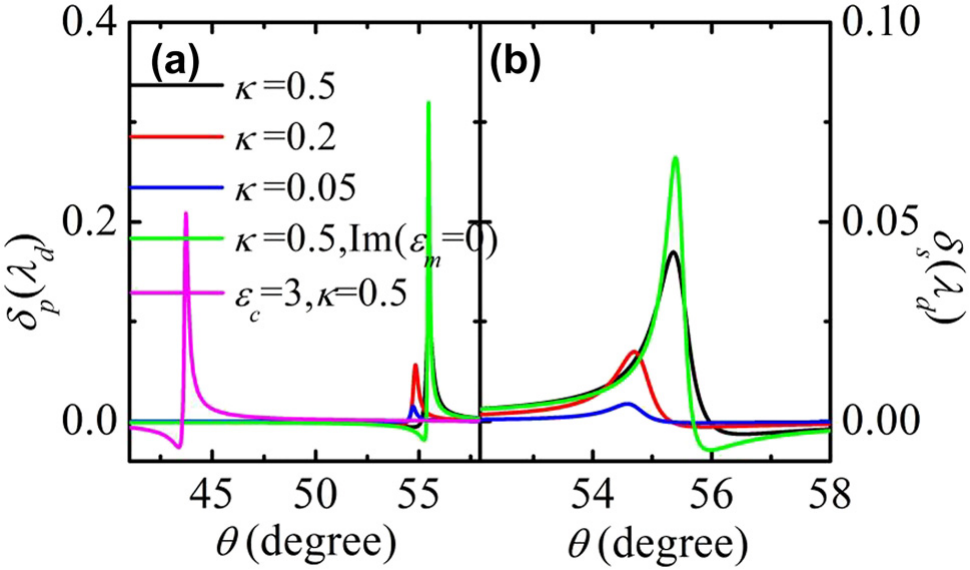
The total Imbert–Fedorov shift of the reflected beams by linearly polarized incident beams for different *κ*. Here, *ɛ*_*c*_ = 4, *d*_*m*_ = 100 nm, and *d*_*c*_ = 150 nm.

**Figure 9:**
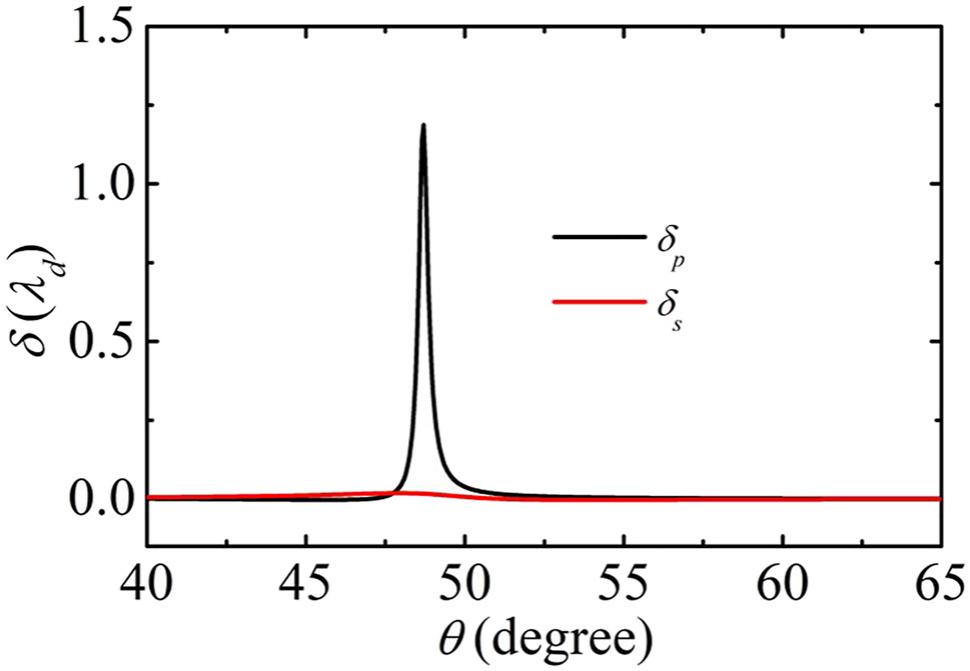
The Imbert–Fedorov shifts of the reflected beams with *d*_*m*_ = 50 nm, *d*_*c*_ = 200 nm, and *κ* = 0.1.

The expressions of *δ*_*ry*_ and *τ* can be found in Section 3 of the Supplementary material. In Eq. (21), we show that the transverse shift is a combination of a *z*_*r*_-independent term and a *z*_*r*_-dependent term. The *z*_*r*_-dependent term can be regarded as a small angler shift inclining from the axis of the beam centroid. Since *r*_*pp*_ and *r*_*ss*_ are weakly dependent on the chirality, the Imbert–Fedorov shift of the reflected beams *δ* = Δ_*ry*_/*k*_*d*_*τ* is proportional to *s*_*x*_ and *κ*. In Figure 8, the Imbert–Fedorov shifts under different *ɛ*_*c*_ and *κ* for TM and TE-polarized incident beams are plotted. Large Imbert–Fedorov shifts happen at the angles satisfying the phase-matching conditions. Since the TM-polarized incident wave has a stronger coupling strength Ω_*p*_ with the SPP, a larger spin–orbit coupling happens and consequently results in a larger Imbert–Fedorov shift. The dissipation in the system can weaken the interaction between the incident waves and the SPP, which induces smaller spin–orbit coupling as well as Imbert–Fedorov shifts. It should be noted that if there are no SPPs on the interfaces between the metal and the chiral material, the reflection of the incident beam is approximately the same as that the beam incident on a semi-infinite metal. The spin–orbit coupling is weak and the transverse spatial shift of a circularly polarized incident beam is at the scale of nanometers [30]. The SPP can enhance the spin–orbit coupling efficiently. Similar to the conventional spin Hall effect, in the whole process, the angular momentum perpendicular to the interface is conserved (see Section 4 of the Supplementary material). To enhance the spin–orbit coupling, thin metal thickness *d*_*m*_ can be adopted. For instance, as shown in Figure 9, when *d*_*m*_ = 50 nm, the spin–orbit coupling, and consequently the Imbert–Fedorov shift, can be enlarged obviously.

## 4 Conclusions

In conclusion, we have investigated the spin of a SPP on a multilayered sandwich configuration and subsequently, the plasmonic spin–assisted spin–orbit coupling induced Imbert–Fedorov shift of linearly polarized beams. The spin of SPP has both transverse and longitudinal terms, and the longitudinal spin AM is determined by the chirality of the chiral material. The propagation of light beams in the plasmonic structure can be analogous to the transport of a single photon in a waveguide QED system, where SPP is analogous to a tunable chiral scatter [53]. The excitation and radiation of the SPP enhance the coupling between the spin AM and extrinsic orbital AM and a large Imbert–Fedorov shift arises for a linearly polarized reflected beam. All the properties of the SPP as well as the spin–orbit coupling can be characterized by the observable reflectivities and transverse spatial shifts. The present investigation provides a platform to study the plasmonic spin as well as plasmonic spin–mediated spin–orbit coupling.

## Supplementary Material

Supplementary Material Details
