# Supplementary material for: Plasmonic spin induced Imbert–Fedorov shift
Source: Nanophotonics. 2023 Feb 15;12(6):1159–67. doi: 10.1515/nanoph-2022-0787 (PMC11501235; doi:10.1515/nanoph-2022-0787)
Supplement: Supplementary file 1 — Supplementary Material Details [file j_nanoph-2022-0787_suppl.pdf]

# Supplementary Material for: Plasmonic spin induced Imbert-Fedorov shift

Hao You[1], Abdullah Alturki[2], Xiaodong Zeng[1*], and M. Suhail Zubairy[2*]
[1]*Department of Physics, Shanghai University, Shanghai 200444, China*
[2]*Institute for Quantum Science and Engineering (IQSE) and Department of Physics and Astronomy, Texas A&M University, College Station, Texas 77843-4242, USA*
*∗Corresponding author: zengxdgood@shu.edu.com, zubairy@physics.tamu.edu*

## I. THE DERIVATION OF THE DISPERSION RELATION OF SURFACE PLASMON POLARIZATION (SPP)

As shown in Fig. 1(a) in the main text, the continuity of the electric and magnetic fields along $x$ and $y$ directions on the upper interface yield:

$$\begin{pmatrix} z_d & z_d & -z_d & -z_d \\ i & -i & i & -i \\ iz_d\sqrt{\varepsilon_d} & -iz_d\sqrt{\varepsilon_d} & -iz_d\sqrt{\varepsilon_d} & iz_d\sqrt{\varepsilon_d} \\ -\sqrt{\varepsilon_d} & -\sqrt{\varepsilon_d} & -\sqrt{\varepsilon_d} & -\sqrt{\varepsilon_d} \end{pmatrix} \begin{pmatrix} a_{1i} \\ b_{1i} \\ a_{1r} \\ b_{1r} \end{pmatrix} = \begin{pmatrix} z_m & z_m & -z_m & -z_m \\ i & -i & i & -i \\ iz_m\sqrt{\varepsilon_m} & -iz_m\sqrt{\varepsilon_m} & -iz_m\sqrt{\varepsilon_m} & iz_m\sqrt{\varepsilon_m} \\ -\sqrt{\varepsilon_m} & -\sqrt{\varepsilon_m} & -\sqrt{\varepsilon_m} & -\sqrt{\varepsilon_m} \end{pmatrix} \begin{pmatrix} a_{2i} \\ b_{2i} \\ a_{2r} \\ b_{2r} \end{pmatrix}, \tag{S1}$$

where $z_j = \beta_j/k_0$, $\beta_j = \sqrt{k_j^2 - k_x^2}$, $k_j = n_j k_0 (j = d, m)$ are the wave numbers in the corresponding mediums, $n_j$ is the index, $k_x$ are the $x$ components of $k_j$, $\varepsilon_d$ and $\varepsilon_m$ are the relative permittivity of the prism and the metal, and $a_{ji(r)}$ and $b_{ji(r)}$ are the right- and left-circularly polarized components of incident (reflected) beam in the $j$th medium, respectively. Similarly, on the second interface from the top,

$$\begin{pmatrix} z_m e^{-i\beta_m d_m} & z_m e^{-i\beta_m d_m} & -z_m e^{i\beta_m d_m} & -z_m e^{i\beta_m d_m} \\ ie^{-i\beta_m d_m} & -ie^{-i\beta_m d_m} & ie^{i\beta_m d_m} & -ie^{i\beta_m d_m} \\ iz_m\sqrt{\varepsilon_m}e^{-i\beta_m d_m} & -iz_m\sqrt{\varepsilon_m}e^{-i\beta_m d_m} & -iz_m\sqrt{\varepsilon_m}e^{i\beta_m d_m} & iz_m\sqrt{\varepsilon_m}e^{i\beta_m d_m} \\ -\sqrt{\varepsilon_m}e^{-i\beta_m d_m} & -\sqrt{\varepsilon_m}e^{-i\beta_m d_m} & -\sqrt{\varepsilon_m}e^{i\beta_m d_m} & -\sqrt{\varepsilon_m}e^{i\beta_m d_m} \end{pmatrix} \begin{pmatrix} a_{2i} \\ b_{2i} \\ a_{2r} \\ b_{2r} \end{pmatrix} = \begin{pmatrix} z_+ & z_- & -z_+ & -z_- \\ i & -i & i & -i \\ iz_+\sqrt{\varepsilon_c} & -iz_-\sqrt{\varepsilon_c} & -iz_+\sqrt{\varepsilon_c} & iz_-\sqrt{\varepsilon_c} \\ -\sqrt{\varepsilon_c} & -\sqrt{\varepsilon_c} & -\sqrt{\varepsilon_c} & -\sqrt{\varepsilon_c} \end{pmatrix} \begin{pmatrix} a_{3i} \\ b_{3i} \\ a_{3r} \\ b_{3r} \end{pmatrix}, \tag{S2}$$

where $d_m$ is the thickness of the metal layer, $z_\pm = \beta_\pm/k_\pm$, $\beta_\pm = \sqrt{k_\pm^2 - k_x^2}$, $k_\pm = n_\pm k_0 = (\sqrt{\varepsilon_c\mu_c} \pm \kappa)k_0$ are the wave numbers of the right-and left-circularly polarized waves in the chiral material. On the third interface from the top,

$$\begin{pmatrix} z_+ e^{-i\beta_+ d_c} & z_- e^{-i\beta_- d_c} & -z_+ e^{i\beta_+ d_c} & -z_- e^{i\beta_- d_c} \\ ie^{-i\beta_+ d_c} & -ie^{-i\beta_- d_c} & ie^{i\beta_+ d_c} & -ie^{i\beta_- d_c} \\ iz_+\sqrt{\varepsilon_c}e^{-i\beta_+ d_c} & -iz_-\sqrt{\varepsilon_c}e^{-i\beta_- d_c} & -iz_+\sqrt{\varepsilon_c}e^{i\beta_+ d_c} & iz_-\sqrt{\varepsilon_c}e^{i\beta_- d_c} \\ -\sqrt{\varepsilon_c}e^{-i\beta_+ d_c} & -\sqrt{\varepsilon_c}e^{-i\beta_- d_c} & -\sqrt{\varepsilon_c}e^{i\beta_+ d_c} & -\sqrt{\varepsilon_c}e^{i\beta_- d_c} \end{pmatrix} \begin{pmatrix} a_{3i} \\ b_{3i} \\ a_{3r} \\ b_{3r} \end{pmatrix} = \begin{pmatrix} z_m & z_m & -z_m & -z_m \\ i & -i & i & -i \\ iz_m\sqrt{\varepsilon_m} & -iz_m\sqrt{\varepsilon_m} & -iz_m\sqrt{\varepsilon_m} & iz_m\sqrt{\varepsilon_m} \\ -\sqrt{\varepsilon_m} & -\sqrt{\varepsilon_m} & -\sqrt{\varepsilon_m} & -\sqrt{\varepsilon_m} \end{pmatrix} \begin{pmatrix} a_{4i} \\ b_{4i} \\ a_{4r} \\ b_{4r} \end{pmatrix}. \tag{S3}$$

Finally, the continuity equations at the bottom interface are given by

$$\begin{pmatrix} z_m e^{-i\beta_m d_m} & z_m e^{-i\beta_m d_m} & -z_m e^{i\beta_m d_m} & -z_m e^{i\beta_m d_m} \\ ie^{-i\beta_m d_m} & -ie^{-i\beta_m d_m} & ie^{i\beta_m d_m} & -ie^{i\beta_m d_m} \\ iz_m\sqrt{\varepsilon_m}e^{-i\beta_m d_m} & -iz_m\sqrt{\varepsilon_m}e^{-i\beta_m d_m} & -iz_m\sqrt{\varepsilon_m}e^{i\beta_m d_m} & iz_m\sqrt{\varepsilon_m}e^{i\beta_m d_m} \\ -\sqrt{\varepsilon_m}e^{-i\beta_m d_m} & -\sqrt{\varepsilon_m}e^{-i\beta_m d_m} & -\sqrt{\varepsilon_m}e^{i\beta_m d_m} & -\sqrt{\varepsilon_m}e^{i\beta_m d_m} \end{pmatrix} \begin{pmatrix} a_{4i} \\ b_{4i} \\ a_{4r} \\ b_{4r} \end{pmatrix} = \begin{pmatrix} z_d & z_d & -z_d & -z_d \\ i & -i & i & -i \\ iz_d\sqrt{\varepsilon_d} & -iz_d\sqrt{\varepsilon_d} & -iz_d\sqrt{\varepsilon_d} & iz_d\sqrt{\varepsilon_d} \\ -\sqrt{\varepsilon_d} & -\sqrt{\varepsilon_d} & -\sqrt{\varepsilon_d} & -\sqrt{\varepsilon_d} \end{pmatrix} \begin{pmatrix} a_{5i} \\ b_{5i} \\ a_{5r} \\ b_{5r} \end{pmatrix}. \tag{S4}$$

The reflected and transmitted fields for an incident beam with any polarization can be obtained from Eqs. (S1-S4). For instance, for a TM-polarized incident wave, $a_{1i} = b_{1i} = 0.5$. The reflection field contains TM and TE-polarized components with amplitudes

$$r_{pp} = a_{1r} + b_{1r} = \frac{rpp}{fm}, \tag{S5}$$

and

$$r_{sp} = i(a_{1r} - b_{1r}) = \frac{rsp}{fm}. \tag{S6}$$

The reflection coefficients are

$$\begin{aligned} rpp =& c_{14}c_{32}\varepsilon_d - c_{12}c_{34}\varepsilon_d + c_{31}c_{42}z_d^2 - c_{32}c_{41}z_d^2 + c_{11}c_{22}\varepsilon_d z_d^2 - c_{12}c_{21}\varepsilon_d z_d^2 \\ &+ c_{12}c_{24}\varepsilon_d^{3/2}z_d - c_{14}c_{22}\varepsilon_d^{3/2}z_d - c_{11}c_{32}\varepsilon_d^{1/2}z_d + c_{11}c_{33}\varepsilon_d z_d^2 + c_{12}c_{31}\varepsilon_d^{1/2}z_d \\ &- c_{13}c_{31}\varepsilon_d z_d^2 + c_{13}c_{34}\varepsilon_d^{3/2}z_d - c_{14}c_{33}\varepsilon_d^{3/2}z_d + c_{21}c_{43}\varepsilon_d z_d^4 - c_{22}c_{44}\varepsilon_d z_d^2 \\ &- c_{23}c_{41}\varepsilon_d z_d^4 + c_{24}c_{42}\varepsilon_d z_d^2 + c_{32}c_{44}\varepsilon_d^{1/2}z_d - c_{33}c_{44}\varepsilon_d z_d^2 - c_{34}c_{42}\varepsilon_d^{1/2}z_d \\ &+ c_{34}c_{43}\varepsilon_d z_d^2 - c_{13}c_{24}\varepsilon_d^2 z_d^2 + c_{14}c_{23}\varepsilon_d^2 z_d^2 - c_{11}c_{23}\varepsilon_d^{3/2}z_d^3 + c_{13}c_{21}\varepsilon_d^{3/2}z_d^3 \\ &- c_{21}c_{42}\varepsilon_d^{1/2}z_d^3 + c_{22}c_{41}\varepsilon_d^{1/2}z_d^3 + c_{23}c_{44}\varepsilon_d^{3/2}z_d^3 - c_{24}c_{43}\varepsilon_d^{3/2}z_d^3, \\ &- c_{31}c_{43}\varepsilon_d^{1/2}z_d^3 + c_{33}c_{41}\varepsilon_d^{1/2}z_d^3, \end{aligned} \tag{S7}$$

$$\begin{aligned} fm =& c_{12}c_{34}\varepsilon_d - c_{14}c_{32}\varepsilon_d + c_{31}c_{42}z_d^2 - c_{32}c_{41}z_d^2 + c_{11}c_{22}\varepsilon_d z_d^2 - c_{12}c_{21}\varepsilon_d z_d^2 \\ &- c_{12}c_{24}\varepsilon_d^{3/2}z_d + c_{14}c_{22}\varepsilon_d^{3/2}z_d - c_{11}c_{32}\varepsilon_d^{1/2}z_d + c_{11}c_{33}\varepsilon_d z_d^2 + c_{12}c_{31}\varepsilon_d^{1/2}z_d \\ &- c_{13}c_{31}\varepsilon_d z_d^2 - c_{13}c_{34}\varepsilon_d^{3/2}z_d + c_{14}c_{33}\varepsilon_d^{3/2}z_d + c_{21}c_{43}\varepsilon_d z_d^4 + c_{22}c_{44}\varepsilon_d z_d^2 \\ &- c_{23}c_{41}\varepsilon_d z_d^4 - c_{24}c_{42}\varepsilon_d z_d^2 - c_{32}c_{44}\varepsilon_d^{1/2}z_d + c_{33}c_{44}\varepsilon_d z_d^2 + c_{34}c_{42}\varepsilon_d^{1/2}z_d \\ &- c_{34}c_{43}\varepsilon_d z_d^2 + c_{13}c_{24}\varepsilon_d^2 z_d^2 - c_{14}c_{23}\varepsilon_d^2 z_d^2 - c_{11}c_{23}\varepsilon_d^{3/2}z_d^3 + c_{13}c_{21}\varepsilon_d^{3/2}z_d^3 \\ &- c_{21}c_{42}\varepsilon_d^{1/2}z_d^3 + c_{22}c_{41}\varepsilon_d^{1/2}z_d^3 - c_{23}c_{44}\varepsilon_d^{3/2}z_d^3 + c_{24}c_{43}\varepsilon_d^{3/2}z_d^3, \\ &- c_{31}c_{43}\varepsilon_d^{1/2}z_d^3 + c_{33}c_{41}\varepsilon_d^{1/2}z_d^3, \end{aligned} \tag{S8}$$

$$\begin{aligned} rsp =& 2c_{14}c_{31}\varepsilon_d z_d - 2c_{11}c_{34}\varepsilon_d z_d - 2c_{21}c_{44}\varepsilon_d z_d^3 + 2c_{24}c_{41}\varepsilon_d z_d^3 + 2c_{11}c_{24}\varepsilon_d^{3/2}z_d^2 \\ &- 2c_{14}c_{21}\varepsilon_d^{3/2}z_d^2 + 2c31c44\varepsilon_d^{1/2}z_d^2 - 2c_{34}c_{41}\varepsilon_d^{1/2}z_d^2, \end{aligned} \tag{S9}$$

where

$$\begin{aligned} c_{11} =& ((\frac{z_m}{z_+}J_+ + \frac{z_m}{z_-}J_-)\frac{\sqrt{\varepsilon_c}}{\sqrt{\varepsilon_m}} + (\frac{z_+}{\sqrt{\varepsilon_c}}J_+ + \frac{z_-}{\sqrt{\varepsilon_c}}J_-)\frac{\sqrt{\varepsilon_m}}{\sqrt{\varepsilon_c}})M_+M_- \\ &+ (A_- + A_+)(M_+^2 + M_-^2), \end{aligned} \tag{S10}$$

$$c_{12} =(z_+J_+ - z_-J_-)iM_+^2 - (A_- - A_+)\frac{i\sqrt{\varepsilon_c}z_m}{\sqrt{\varepsilon_m}}M_+M_- + (A_+ - A_-)\frac{i\sqrt{\varepsilon_m}z_m}{\sqrt{\varepsilon_c}}M_+M_- + (\frac{z_m}{z_+}J_+ - \frac{z_m}{z_-}J_-)\frac{i\sqrt{\varepsilon_m}z_m}{\sqrt{\varepsilon_c}}M_-^2, \tag{S11}$$

$$c_{13} =- (z_+J_+ - z_-J_-)\frac{i}{\sqrt{\varepsilon_m}z_m}M_+M_- - (A_+ - A_-)(\frac{i\sqrt{\varepsilon_c}}{\varepsilon_m}M_-^2 + \frac{i}{\sqrt{\varepsilon_c}}M_+^2) - (\frac{z_m}{z_+}J_+ - \frac{z_m}{z_-}J_-)\frac{i}{\sqrt{\varepsilon_c}}M_+M_-, \tag{S12}$$

$$c_{14} =2(A_- + A_+)\frac{z_m}{\sqrt{\varepsilon_m}}M_+M_- + (\frac{\sqrt{\varepsilon_c}}{z_-}J_- + \frac{\sqrt{\varepsilon_c}}{z_+}J_+)\frac{z_m^2}{\varepsilon_m}M_-^2 + (\frac{z_-}{\sqrt{\varepsilon_c}}J_- + \frac{z_+}{\sqrt{\varepsilon_c}}J_+)M_+^2, \tag{S13}$$

$$c_{21} =- (\frac{i}{z_+}J_+ - \frac{i}{z_-}J_-)M_+^2 - (A_+ - A_-)\frac{i\sqrt{\varepsilon_c}}{\sqrt{\varepsilon_m}z_m}M_+M_- - (A_+ - A_-)\frac{i\sqrt{\varepsilon_m}}{\sqrt{\varepsilon_c}z_2}M_+M_- - (z_+J_+ - z_-J_-)\frac{i}{z_m^2}M_-^2, \tag{S14}$$

$$c_{22} =(A_- + A_+)M_+^2 + (\frac{\sqrt{\varepsilon_c}z_-}{\sqrt{\varepsilon_m}z_m}J_- + \frac{\sqrt{\varepsilon_c}z_+}{\sqrt{\varepsilon_m}z_m}J_+)M_+M_- + (\frac{\sqrt{\varepsilon_m}z_m}{\sqrt{\varepsilon_c}z_-}J_- + \frac{\sqrt{\varepsilon_m}z_m}{\sqrt{\varepsilon_c}z_+}J_+)M_+M_- + (A_- + A_+)M_-^2, \tag{S15}$$

$$c_{23} =- (\frac{\sqrt{\varepsilon_c}z_-}{\varepsilon_m z_m^2}J_- + \frac{\sqrt{\varepsilon_c}z_+}{\varepsilon_m z_m^2}J_+)M_-^2 - (A_- + A_+)\frac{1}{\sqrt{\varepsilon_m}z_m}M_+M_- - (\frac{1}{\sqrt{\varepsilon_c}z_-}J_- + \frac{1}{\sqrt{\varepsilon_c}z_+}J_+)M_+^2 - (A_- + A_+)\frac{1}{\sqrt{\varepsilon_m}z_m}M_+M_-, \tag{S16}$$

$$c_{24} =- (\frac{i}{z_+}J_+ - \frac{i}{z_-}J_-)\frac{z_m}{\sqrt{\varepsilon_m}}M_+M_- - (A_+ - A_-)\frac{i\sqrt{\varepsilon_c}}{\varepsilon_m}M_-^2 - (A_+ - A_-)\frac{i}{\sqrt{\varepsilon_c}}M_+^2 - (z_+J_+ - z_-J_-)\frac{i}{\sqrt{\varepsilon_m}z_m}M_+M_-, \tag{S17}$$

$$c_{31} =- (\frac{z_m}{z_-}J_- - \frac{z_m}{z_+}J_+)i\sqrt{\varepsilon_m}M_+M_- - (A_- - A_+)i\sqrt{\varepsilon_c}M_+^2 - (A_- - A_+)\frac{i\varepsilon_m}{\sqrt{\varepsilon_c}}M_-^2 - (z_-J_- - z_+J_+)\frac{i\sqrt{\varepsilon_m}}{z_m}M_+M_-, \tag{S18}$$

$$c_{32} =- (A_+ + A_-)\sqrt{\varepsilon_m}z_mM_+M_- - (z_-J_- + z_+J_+)\sqrt{\varepsilon_c}M_+^2 - (\frac{z_m}{z_-}J_- + \frac{z_m}{z_+}J_+)\frac{\varepsilon_m z_m}{\sqrt{\varepsilon_c}}M_-^2 - (A_+ + A_-)\sqrt{\varepsilon_m}z_mM_+M_-, \tag{S19}$$

$$c_{33} =(A_- + A_+)M_-^2 + (z_-J_- + z_+J_+)\frac{\sqrt{\varepsilon_c}}{\sqrt{\varepsilon_m}z_m}M_+M_- + (\frac{z_m}{z_-}J_- + \frac{z_m}{z_+}J_+)\frac{\sqrt{\varepsilon_m}}{\sqrt{\varepsilon_c}}M_+M_- + (A_- + A_+)M_+^2, \tag{S20}$$

$$c_{34} = -(\frac{z_m}{z_-}J_- - \frac{z_m}{z_+}J_+)iz_m M_-^2 - (A_- - A_+)i\frac{\sqrt{\varepsilon}_m z_m}{\sqrt{\varepsilon}_c}M_+M_- \\ -(A_- - A_+)i\frac{\sqrt{\varepsilon}_c z_m}{\sqrt{\varepsilon}_m}M_+M_- - (z_-J_- - z_+J_+)iM_+^2, \tag{S21}$$

$$c_{41} = (A_+ + A_-)\frac{\sqrt{\varepsilon}_m}{z_m}M_+M_- - (\frac{\sqrt{\varepsilon}_c}{z_-}J_- - \frac{\sqrt{\varepsilon}_c}{z_+}J_+)M_+^2 \\ -(\frac{z_-}{\sqrt{\varepsilon}_c}J_- - \frac{z_+}{\sqrt{\varepsilon}_c}J_+)\frac{\varepsilon_m}{z_m^2}M_-^2 + (A_+ + A_-)\frac{\sqrt{\varepsilon}_m}{z_2}M_+M_-, \tag{S22}$$

$$c_{42} = -(z_-J_- - z_+J_+)i\frac{\sqrt{\varepsilon}_m}{z_m}M_+M_- - (A_- - A_+)i\sqrt{\varepsilon}_c M_+^2 \\ +(A_+ - A_-)i\frac{\varepsilon_m}{\sqrt{\varepsilon}_c}M_-^2 + (\frac{1}{z_+}J_+ - \frac{1}{z_-}J_-)i\sqrt{\varepsilon}_m z_m M_+M_-, \tag{S23}$$

$$c_{43} = -(z_+J_+ - z_-J_-)\frac{i}{z_m^2}M_-^2 - (A_+ - A_-)i\frac{\sqrt{\varepsilon}_c}{\sqrt{\varepsilon}_m z_m}M_+M_- \\ -(A_+ - A_-)i\frac{\sqrt{\varepsilon}_m}{\sqrt{\varepsilon}_c z_m}M_+M_- - (\frac{1}{z_+}J_+ - \frac{1}{z_-}J_-)iM_+^2, \tag{S24}$$

$$c_{44} = (A_+ + A_-)M_-^2 + (\frac{\sqrt{\varepsilon}_c}{z_-}J_- + \frac{\sqrt{\varepsilon}_c}{z_+}J_+)\frac{z_2}{\sqrt{\varepsilon}_m}M_+M_- \\ +(\frac{z_-}{\sqrt{\varepsilon}_c}J_- + \frac{z_+}{\sqrt{\varepsilon}_c}J_+)\frac{\sqrt{\varepsilon}_m}{z_2}M_+M_- + (A_- + A_+)M_+^2. \tag{S25}$$

Here $M_+ = (e^{i\beta_m d} + e^{-i\beta_m d})/2$, $M_- = (e^{i\beta_m d} - e^{-i\beta_m d})/2$, $A_- = (e^{i\beta_- d_c} + e^{-i\beta_- d_c})/4$, $A_+ = (e^{i\beta_+ d_c} + e^{-i\beta_+ d_c})/4$, $J_- = (e^{i\beta_- d_c} - e^{-i\beta_- d_c})/4$, and $J_+ = (e^{i\beta_+ d_c} - e^{-i\beta_+ d_c})/4$.

The dispersion relation of the SPP can be expressed as follows (the denominator of the reflection coefficient is equal to 0):

$$z_d^2(B_i e^{-i\beta_+ d_c} + B_r e^{i\beta_+ d_c})^2 - z_d^2(Ce^{-i\beta_- d_c} + Ce^{i\beta_- d_c})^2 \\ = z_+^2(B_i e^{-i\beta_+ d_c} + B_r e^{i\beta_+ d_c})^2 - z_-^2(Ce^{-i\beta_- d_c} + Ce^{i\beta_- d_c})^2. \tag{S26}$$

Here

$$B_i = \frac{r_1(\sqrt{\varepsilon}_c z_+ - \sqrt{\varepsilon}_d z_d) + r_2(\sqrt{\varepsilon}_d z_+ + \sqrt{\varepsilon}_c z_d) + (\sqrt{\varepsilon}_d z_+ + \sqrt{\varepsilon}_c)}{4\sqrt{\varepsilon}_c z_+}, \tag{S27}$$

$$B_r = \frac{r_1(\sqrt{\varepsilon}_c z_+ + \sqrt{\varepsilon}_d z_d) + r_2(\sqrt{\varepsilon}_d z_+ - \sqrt{\varepsilon}_c z_d) + (\sqrt{\varepsilon}_d z_+ - \sqrt{\varepsilon}_c)}{4\sqrt{\varepsilon}_c z_+}, \tag{S28}$$

$$C = \frac{r_1\sqrt{\varepsilon}_c - r_2\sqrt{\varepsilon}_d - \sqrt{\varepsilon}_d}{-4\sqrt{\varepsilon}_c} + \frac{r_1\sqrt{\varepsilon}_d z_d + r_2\sqrt{\varepsilon}_c z_d + \sqrt{\varepsilon}_c}{4\sqrt{\varepsilon}_c z_-}, \tag{S29}$$

with

$$
\begin{aligned}
r_1 =\Big[&(M_+ z_d + M_- z_m \frac{\sqrt{\varepsilon_d}}{\sqrt{\varepsilon_m}} - M_- z_+ \frac{\sqrt{\varepsilon_m} z_d}{\sqrt{\varepsilon_c} z_m} - M_+ z_+ \frac{\sqrt{\varepsilon_d}}{\sqrt{\varepsilon_c}})\\
&(-M_- \frac{\sqrt{\varepsilon_m} z_d}{\sqrt{\varepsilon_c} z_m} + M_+ \frac{\sqrt{\varepsilon_d}}{\sqrt{\varepsilon_c}} + M_+ \frac{z_d}{z_-} - M_- \frac{\sqrt{\varepsilon_d} z_m}{\sqrt{\varepsilon_m} z_-})\\
&-(-M_+ z_d + M_- z_m \frac{\sqrt{\varepsilon_d}}{\sqrt{\varepsilon_m}} + M_- z_+ \frac{\sqrt{\varepsilon_m} z_d}{\sqrt{\varepsilon_c} z_m} - M_+ z_+ \frac{\sqrt{\varepsilon_d}}{\sqrt{\varepsilon_c}})\\
&(M_- \frac{\sqrt{\varepsilon_m} z_d}{\sqrt{\varepsilon_c} z_m} + M_+ \frac{\sqrt{\varepsilon_d}}{\sqrt{\varepsilon_c}} - M_+ \frac{z_d}{z_-} - M_- \frac{\sqrt{\varepsilon_d} z_m}{\sqrt{\varepsilon_m} z_-})\Big]\\
&/\Big[(M_+ z_+ - M_- z_m \frac{\sqrt{\varepsilon_m}}{\sqrt{\varepsilon_c}} - M_- z_+ \frac{\sqrt{\varepsilon_d} z_d}{\sqrt{\varepsilon_m} z_m} + M_+ z_d \frac{\sqrt{\varepsilon_d}}{\sqrt{\varepsilon_c}})\\
&(-M_- \frac{\sqrt{\varepsilon_m} z_d}{\sqrt{\varepsilon_c} z_m} + M_+ \frac{\sqrt{\varepsilon_d}}{\sqrt{\varepsilon_c}} + M_+ \frac{z_d}{z_-} - M_- \frac{\sqrt{\varepsilon_d} z_m}{\sqrt{\varepsilon_m} z_-})\\
&-(-M_+ z_d + M_- z_m \frac{\sqrt{\varepsilon_d}}{\sqrt{\varepsilon_m}} + M_- z_+ \frac{\sqrt{\varepsilon_m} z_d}{\sqrt{\varepsilon_c} z_m} - M_+ z_+ \frac{\sqrt{\varepsilon_d}}{\sqrt{\varepsilon_c}})\\
&(M_+ - M_- \frac{\sqrt{\varepsilon_d} z_d}{\sqrt{\varepsilon_m} z_m} - M_- \frac{\sqrt{\varepsilon_m} z_m}{\sqrt{\varepsilon_c} z_-} + M_+ \frac{\sqrt{\varepsilon_d} z_d}{\sqrt{\varepsilon_c} z_-})\Big],
\end{aligned}
\tag{S30}
$$

and

$$
\begin{aligned}
r_2 =\Big[&(M_+ z_d + M_- z_m \frac{\sqrt{\varepsilon_d}}{\sqrt{\varepsilon_m}} - M_- z_+ \frac{\sqrt{\varepsilon_m} z_d}{\sqrt{\varepsilon_c} z_m} - M_+ z_+ \frac{\sqrt{\varepsilon_d}}{\sqrt{\varepsilon_c}})\\
&(M_+ - M_- \frac{\sqrt{\varepsilon_d} z_d}{\sqrt{\varepsilon_m} z_m} - M_- \frac{\sqrt{\varepsilon_m} z_m}{\sqrt{\varepsilon_c} z_-} + M_+ \frac{\sqrt{\varepsilon_d} z_d}{\sqrt{\varepsilon_c} z_-})\\
&-(M_+ z_+ - M_- z_+ \frac{\sqrt{\varepsilon_d} z_d}{\sqrt{\varepsilon_m} z_m} - M_- z_m \frac{\sqrt{\varepsilon_m}}{\sqrt{\varepsilon_c}} + M_+ z_d \frac{\sqrt{\varepsilon_d}}{\sqrt{\varepsilon_c}})\\
&(M_- \frac{\sqrt{\varepsilon_m} z_d}{\sqrt{\varepsilon_c} z_m} + M_+ \frac{\sqrt{\varepsilon_d}}{\sqrt{\varepsilon_c}} - M_+ \frac{z_d}{z_-} - M_- \frac{\sqrt{\varepsilon_d} z_m}{\sqrt{\varepsilon_m} z_-})\Big]\\
&/\Big[(M_+ z_+ - M_- z_m \frac{\sqrt{\varepsilon_m}}{\sqrt{\varepsilon_c}} - M_- z_+ \frac{\sqrt{\varepsilon_d} z_d}{\sqrt{\varepsilon_m} z_m} + M_+ z_d \frac{\sqrt{\varepsilon_d}}{\sqrt{\varepsilon_c}})\\
&(-M_- \frac{\sqrt{\varepsilon_m} z_d}{\sqrt{\varepsilon_c} z_m} + M_+ \frac{\sqrt{\varepsilon_d}}{\sqrt{\varepsilon_c}} + M_+ \frac{z_d}{z_-} - M_- \frac{\sqrt{\varepsilon_d} z_m}{\sqrt{\varepsilon_m} z_-})\\
&-(-M_+ z_d + M_- z_m \frac{\sqrt{\varepsilon_d}}{\sqrt{\varepsilon_m}} + M_- z_+ \frac{\sqrt{\varepsilon_m} z_d}{\sqrt{\varepsilon_c} z_m} - M_+ z_+ \frac{\sqrt{\varepsilon_d}}{\sqrt{\varepsilon_c}})\\
&(M_+ - M_- \frac{\sqrt{\varepsilon_d} z_d}{\sqrt{\varepsilon_m} z_m} - M_- \frac{\sqrt{\varepsilon_m} z_m}{\sqrt{\varepsilon_c} z_-} + M_+ \frac{\sqrt{\varepsilon_d} z_d}{\sqrt{\varepsilon_c} z_-})\Big].
\end{aligned}
\tag{S31}
$$

## II. ADDITIONAL FIGURES OF THE FIELD DISTRIBUTIONS

The electric field distribution of the asymmetric mode in the structure as shown in Fig. 1(a) is presented in Fig. S1. Besides, the electric field distribution of the symmetric mode of a chiral material slab embedded in infinite metal is presented in Fig. S2.

## III. THE IMBERT-FEDOROV SHIFT

In paraxial optics, the incident field of an arbitrarily polarized beam can be written as [1]

$$
\mathbf{E}_i(x_i, y_i, z_i) = (f_p \hat{\mathbf{e}}_{ix} + f_s \hat{\mathbf{e}}_{iy}) \exp\Big(-\frac{k_d}{2}\frac{x_i^2 + y_i^2}{Z_R + i z_r}\Big), \tag{S32}
$$

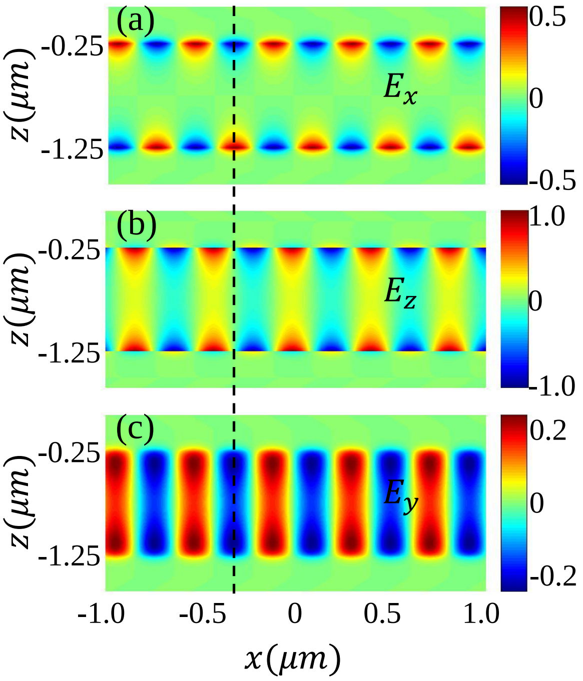


Fig. S1. The electric field distribution of the asymmetric mode for all three directions. The parameters are the same as in Fig. 3 in the main text.

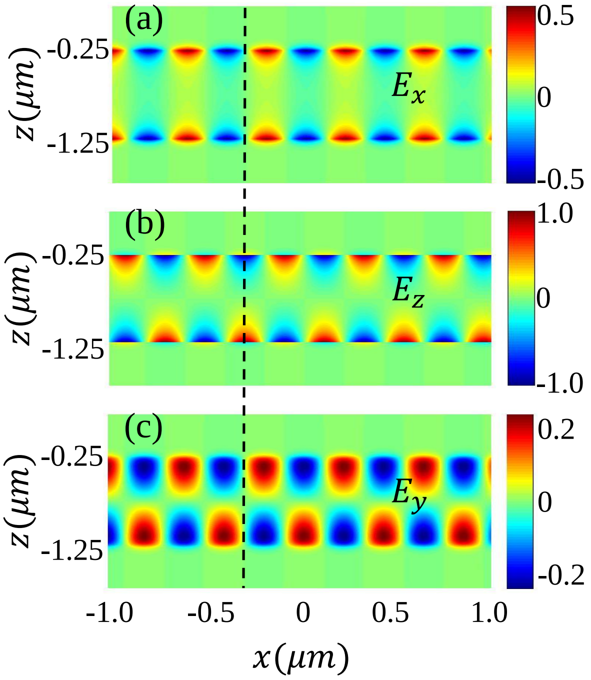


Fig. S2. The electric field distribution of the symmetric mode for all three directions with a structure of a chiral material slab embedded in infinite metal.

where $Z_R = k_d W^2/2$ is the Rayleigh length and $W$ is the Gaussian beam's minimum waist. The complex-valued vectors $f_p$ and $f_s$ determine the polarizations of the incident beam. The reflected field can be solved by employing Fourier transformations, i.e.,

$$\mathbf{E}_r(x_r, y_r, z_r) = \int \mathbf{E}_r(k_{rx}, k_{ry}) \exp[i(k_{rx}x_r + k_{ry}y_r + k_{rz}z_r)]dk_{rx}dk_{ry}, \tag{S33}$$

where $k_{rx} = -k_{ix}$, and $k_{ry} = k_{iy}$ are the components of the wave vector of the reflected beams. In the paraxial approximation, $k_{rz} \approx -(k_{rx}^2 + k_{ry}^2)/2k_d$. The reflected angular spectrum $\mathbf{E}_r(k_{rx}, k_{ry})$, which is related to the boundary distribution of the electric field, can then be expressed as:

$$\begin{pmatrix} E_{rp} \\ E_{rs} \end{pmatrix} = \begin{pmatrix} r_{pp} + \frac{k_{ry}}{k_d}(r_{sp} - r_{ps})cot\theta & r_{ps} + \frac{k_{ry}}{k_d}(r_{pp} + r_{ss})cot\theta \\ r_{sp} - \frac{k_{ry}}{k_d}(r_{pp} + r_{ss})cot\theta & r_{ss} + \frac{k_{ry}}{k_d}(r_{sp} - r_{ps})cot\theta \end{pmatrix} \begin{pmatrix} f_p \\ f_s \end{pmatrix}$$
$$\times \exp\Big[-\frac{Z_R(k_{rx}^2 + k_{ry}^2)}{2k_d}\Big]. \tag{S34}$$

Here $r_{ab}$ is a function of $k_{ix}$, $k_{iy}$ and the incident angle $\theta$, and $a(b) = s, p$. In the linear approximation, it can be written as:

$$\begin{aligned} r_{ab}(k_{ix}, k_{iy}) &= r_{ab}(0,0) + k_{ix}\frac{\partial r_{ab}(k_{ix}, k_{iy})}{\partial k_{ix}}\Big|_{k_{ix}=0, k_{iy}=0} \\ &= r_{ab}(0,0) + k_{ix}\frac{\partial r_{ab}(0,0)}{\partial \theta}, \end{aligned} \tag{S35}$$

where $r_{ab}(0,0)$, which only depends on $\theta$, is the ordinary reflection coefficient with respect to the central wave vector of the incident beam.

Inserting Eq. (S35) into Eq. (S34), the reflected fields in the prism at $z_r > 0$ can be expressed as:

$$\begin{aligned} E_{rp}(x_r, y_r) =& \Big[r_{pp}\Big(1 - i\frac{x_r}{Z_R + iz_r}\frac{\partial}{\partial\theta}\ln r_{pp}\Big)f_p + r_{ps}\Big(1 - i\frac{x_r}{Z_R + iz_r}\frac{\partial}{\partial\theta}\ln r_{ps}\Big)f_s \\ &+ i\frac{y_r}{Z_R + iz_r}(r_{sp} - r_{ps})f_p\cot\theta + i\frac{y_r}{Z_R + iz_r}(r_{pp} + r_{ss})f_s\cot\theta\Big] \\ &\times \exp\Big[-\frac{k_d(x_r^2 + y_r^2)}{2(Z_R + iz_r)}\Big], \end{aligned} \tag{S36}$$

$$\begin{aligned} E_{rs}(x_r, y_r) =& \Big[r_{sp}\Big(1 - i\frac{x_r}{Z_R + iz_r}\frac{\partial}{\partial\theta}\ln r_{sp}\Big)f_p + r_{ss}\Big(1 - i\frac{x_r}{Z_R + iz_r}\frac{\partial}{\partial\theta}\ln r_{ss}\Big)f_s \\ &- i\frac{y_r}{Z_R + iz_r}(r_{pp} + r_{ss})f_p\cot\theta + i\frac{y_r}{Z_R + iz_r}(r_{sp} - r_{ps})f_s\cot\theta\Big] \\ &\times \exp\Big[-\frac{k_d(x_r^2 + y_r^2)}{2(Z_R + iz_r)}\Big]. \end{aligned} \tag{S37}$$

The beam centroid is given by

$$\langle y_r \rangle = \frac{\iint y_r I(x_r, y_r, z_r)dx_r dy_r}{\iint I(x_r, y_r, z_r)dx_r dy_r}. \tag{S38}$$

Here, $I(x, y, z)$ is the spatial dependent field intensity. It follows on substituting Eqs. (S36) and (S37) into Eq. (S38), and keeping up to the first-order term of $k_y$. The transverse shift of the centroid of the reflected beam can be achieved as

$$\langle y_r \rangle = \frac{\Delta_{ry}}{k_d\tau} + \frac{z_r}{Z_R}\frac{\delta_{ry}}{k_d\tau}, \tag{S39}$$

where

$$\begin{aligned} \Delta_{ry} =& -\cot\theta \times \mathrm{Im}[r_{pp}r_{ps}^* + r_{sp}r_{ss}^* + 2(r_{pp}^* r_{sp}|f_p|^2 + r_{ps}^* r_{ss}|f_s|^2) \\ &- f_p f_s^*(|r_{pp}|^2 + 2r_{pp}r_{ss}^* + |r_{sp}|^2 - 2r_{ps}^* r_{sp} + |r_{ps}|^2 + |r_{ss}|^2)], \end{aligned} \tag{S40}$$

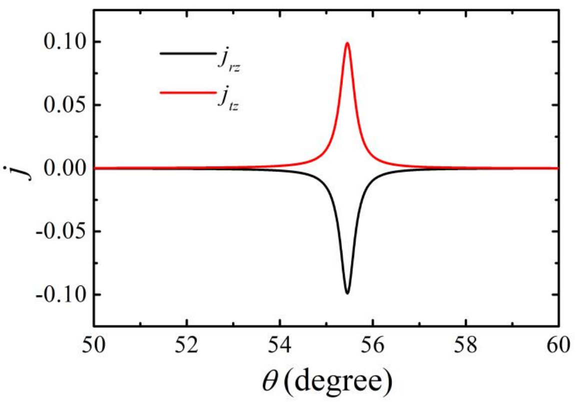


Fig. S3. The total angular momentum of the reflected and transmitted fields.

$$\delta_{ry} = -\cot\theta \times \mathrm{Re}[(r_{pp}r_{ps}^* + r_{sp}r_{ss}^*)(|f_s|^2 - |f_p|^2) + f_p f_s^*(|r_{sp}|^2 - |r_{ss}|^2 + |r_{pp}|^2 - |r_{ps}|^2)], \tag{S41}$$

$$\tau = (|r_{pp}|^2 + |r_{sp}|^2)|f_p|^2 + (|r_{ps}|^2 + |r_{ss}|^2)|f_s|^2 + 2\mathrm{Re}(f_p f_s^*(r_{pp}r_{ps}^* + r_{sp}r_{ss}^*)). \tag{S42}$$

## IV. THE CONSERVATION OF THE TOTAL ANGULAR MOMENTUM (AM)

The total AM per photon can be represented as the sum of the extrinsic orbit AM and intrinsic spin AM:

$$\boldsymbol{j}_r = \langle \boldsymbol{y}_r \rangle \times \boldsymbol{k}_r + \sigma_r \boldsymbol{k}_r / k_r, \tag{S43}$$

$$\boldsymbol{j}_t = \langle \boldsymbol{y}_t \rangle \times \boldsymbol{k}_t + \sigma_t \boldsymbol{k}_t / k_t, \tag{S44}$$

where $\sigma_r$ and $\sigma_t$ are the polarization degrees of the reflected and transmitted beams, which are described by

$$\sigma_r = \frac{2\mathrm{Im}(r_{pp}^* r_{sp}|f_p|^2 + r_{ps}^* r_{sp} f_p f_s^* + r_{pp}^* r_{ss} f_p^* f_s + r_{ps}^* r_{ss}|f_s|^2)}{(|r_{pp}|^2 + |r_{sp}|^2)|f_p|^2 + (|r_{ps}|^2 + |r_{ss}|^2)|f_s|^2 + 2\mathrm{Re}[(r_{pp}r_{ps}^* + r_{sp}r_{ss}^*)f_p f_s^*]}, \tag{S45}$$

$$\sigma_t = \frac{2\mathrm{Im}(t_{pp}^* t_{sp}|f_p|^2 + t_{ps}^* t_{sp} f_p f_s^* + t_{pp}^* t_{ss} f_p^* f_s + t_{ps}^* t_{ss}|f_s|^2)}{(|t_{pp}|^2 + |t_{sp}|^2)|f_p|^2 + (|t_{ps}|^2 + |t_{ss}|^2)|f_s|^2 + 2\mathrm{Re}[(t_{pp}t_{ps}^* + t_{sp}t_{ss}^*)f_p f_s^*]}. \tag{S46}$$

The transverse shifts of the wave packet fulfill the conservation law for the total AM normal to the interface:

$$Q_r j_{rz} + Q_t j_{tz} = j_{iz}, \tag{S47}$$

where $j_{iz} = \sigma\cos\theta$, $Q_r = (|r_{pp}|^2 + |r_{sp}|^2)|f_p|^2 + (|r_{ps}|^2 + |r_{ss}|^2)|f_s|^2 + 2\mathrm{Re}[(r_{pp}r_{ps}^* + r_{sp}r_{ss}^*)f_p f_s^*]$, and $Q_t = (|t_{pp}|^2 + |t_{sp}|^2)|f_p|^2 + (|t_{ps}|^2 + |t_{ss}|^2)|f_s|^2 + 2\mathrm{Re}[(t_{pp}t_{ps}^* + t_{sp}t_{ss}^*)f_p f_s^*]$ are the energy reflection and transmission probabilities, respectively. As shown in Fig. S3, the total AM of the reflected and transmitted fields are zero for a TM-polarized incident beam.

---

[1] H. Wang and X. Zhang, "Unusual spin Hall effect of a light beam in chiral metamaterials," *Phys. Rev. A*, vol. 83, p. 053820, 2011.
